# Management of Chronic Kidney Disease in Morocco: A Cost-of-Illness Study

**DOI:** 10.7759/cureus.40537

**Published:** 2023-06-16

**Authors:** Amina Chrifi Alaoui, Mohamed Elomari, Noura Qarmiche, Omar Kouiri, Basmat Amal Chouhani, Karima El Rhazi, Samira EL Fakir, Tarik Sqalli Houssaini, Nabil Tachfouti

**Affiliations:** 1 Laboratory of Epidemiology, Clinical Research and Community Health, Faculty of Medicine, Pharmacy, and Dentistry, Sidi Mohamed Ben Abdellah University, Fez, MAR; 2 Nephrology, Dialysis, and Transplantation, Hassan II University Hospital, Fez, MAR; 3 Laboratory of Epidemiology and Research in Health Sciences, Faculty of Medicine, Pharmacy, and Dentistry, Sidi Mohamed Ben Abdellah University, Fez, MAR

**Keywords:** study, cost of illness (coi), direct medical cost, morocco, chronic kidney disease

## Abstract

Introduction: Chronic kidney disease (CKD) is a global public health problem. The aim of this study is to estimate the mean annual direct medical cost per patient with CKD before the start of renal replacement therapy (RRT) in Morocco.

Methods: This is a cross-sectional cost-of-illness study, using a prevalence approach among adults with CKD before RRT in a Moroccan university hospital. Information on direct medical costs was collected from the patient’s report and associated costs were estimated according to national tariff/fee catalogues. We computed annual direct medical costs using society perspective. Costs were then estimated and compared according to CKD stages, health insurance categories, and monthly income.

Results: Eighty-eight participants were included; 63.6% of them were female, their mean age was 61.8±14.0 years, and 76.1% were in stages 4 or 5. The estimated annual direct medical cost of CKD was estimated at $ 2008.80 (95%CI 1528.28-2489.31), Hospitalization, diagnosis, and treatment represented the main expenses of the direct medical cost (32.2%, 29.7%, and 32.2%, respectively). The direct medical cost components were not significantly different between CKD stages.

Conclusion: The cost of CKD in Morocco in its early stages is still lower than the cost of RRT, which brings to light the necessity of secondary prevention of CKD to postpone or prevent the progression to end-stage renal disease.

## Introduction

Chronic kidney disease (CKD) is increasingly recognized as a global public health problem; thus, its prevalence ranges between 5% and 13% across countries [1]. The prevalence rates of CKD appear to be increasing globally [2,3] and are likely to increase further as a consequence of aging populations and the increased prevalence of type II diabetes mellitus [4-6]. Systematic analysis of worldwide population-based data shows that the estimated total number of adults with CKD (any stage) was 177.4 million in men and 210.1 million in women in low- and middle-income countries [7].

CKD and end-stage renal disease (ESRD) are associated with substantial healthcare resource use [8]. Thereby, several studies, conducted in high-income countries, targeted the cost of CKD before renal replacement therapy (RRT). The average annual direct medical costs of care per patient in Germany in 2019 was € 8.030 (95%CI: € 7.848-8.212) for stage 3 of CKD, and € 9.760 (95%CI: € 9.266-10.255) for stage 4 of CKD [9]. The overall annual social cost of CKD in Italy between March 2012 and March 2013 was € 1,809,552,398, which represents 0.11 % of the gross domestic product (GDP) [10]. In the United States, in a study conducted between 1996 and 2001 (published in 2004), the total per-patient follow-up costs were United States Dollar ($) 38.764 in stage 2, $ 33.144 in stage 3, and $ 41.928 in stage 4 of CKD [11]. In England, the cost of CKD to the National Health Services (NHS) in 2010 was estimated at £ 1.44-1.45 billion, which is 1.3% of all NHS spending in that year [5]. In middle and low-income countries, a systematic review reported annual cost per patient for hemodialysis ranged between $ 3.424 and $ 42.785 [12]. As far as middle-income countries are concerned, to the best of our knowledge, one study was conducted by Aoun et al. in Lebanon on CKD before the start of RRT, in which they found that the mean cost of non-dialysis CKD was $ 2988.

In Morocco, The burden of noncommunicable diseases is high, causing 75.3% of all deaths [13]. According to the MAREMAR (Maladies Rénales Chroniques au Maroc) study [14], The adjusted prevalence of subjects with estimated glomerular filtration rate (eGFR) <60 ml/minute/1.73 m^3^ was 1.6%, a low percentage compared with the majority of the reported studies (prevalence between 1.7% and 8.1%), and the adjusted prevalence according to the Moroccan population structure was 2.9% [14].

Morocco devotes 6% of its GDP to the health sector. Morocco's health system is financed mainly by direct payment of the households (50%) then taxes (25%), and finally by health insurance (22%) [15]. The provision of healthcare is characterized by a mixture of public, private profit, and private non-profit services. In the context of limited resources of the healthcare system [16], no data are available on the economic burden of CKD before RRT. The main objective of this study is to estimate the mean annual direct medical cost per patient suffering from CKD before the start of RRT in Morocco.

## Materials and methods

This cross-sectional cost-of-illness study was carried out in the Nephrology Department of Hassan II University Hospital, Fez, Morocco, between October 2019 and October 2020. This study was approved by the Fez Hospital-University Ethics Committee (approval number: 26/18). 

Participants

Patients of interest were defined as persons aged 18 years or older, followed for more than one year for a CKD with an eGFR< 60 ml/minute/1.73 m^2^, not dialyzed nor transplanted, and agreeing to participate in the survey.

Sample size

The sample size was estimated at 88 participants and was calculated using the Kirkwood formula [17] as:

N=(Zα/2+Zβ)2*(P(1-P))/e2)

Where Zα/2 = 1.96; Zβ: critical value of the normal distribution at β (for a power of 80%, β= 0.2 and Zβ= 0.84); P: expected CKD proportion in Moroccan population = 2.9% (MAREMAR study [14]); e: standard error around estimated proportion 5% (+/− 0.05).

Data collection

Data were collected from medical records using proforma, including information on sociodemographic data, CKD stage and duration, and health service use for different care-seeking episodes of CKD during the last year.

Data analysis and cost estimation

Data were entered in Excel 2016 (Microsoft Corporation, Redmond, Washington, United States) to enhance data quality. The two data sets were compared to eliminate data entry errors. Consistency and range checks were used to ensure the completeness of data.

Frequency counts and percentages were used to describe patient demographic and socio‐economic variables. Mean values were reported for all cost estimates as a measure of central tendency, and 95% CI and interquartile range (IQR) were reported. A prevalence-based approach was used to calculate the cost of illness [16]. The direct medical cost of CKD management was divided into three categories: (i) Diagnosis, which included all laboratory tests, radiology exams, and specialized acts like biopsies, (ii) CKD treatments, which included treatments bought by the patient and those provided by the hospital including acute hemodialysis, and (iii) Specialized visits, which included hospitalization, day care hospital, and nephrology consultation.

All health services costs (in public and private health facilities) were estimated according to the National Agency for health insurance official prices [18]. Drug cost was estimated using the unit cost, the daily dosage, and the treatment duration.

Cost results are presented in US Dollars ($). Cost in Moroccan Dirhams (MAD) was converted to $ using the following exchange rate: $ 1 = MAD 9.97 (Open Financial Exchange (OFX) monthly average rate, April 2020).

First, the mean annual unadjusted medical cost data per patient have been calculated by type of cost component in order to estimate the burden of each cost item on the overall direct medical cost. Then the components were compared between CKD stages (stage 3 was defined as an eGFR between 30 and 59 ml/minute/m^2^, stage 4 was defined as an eGFR between 15 and 29 ml/minute/m^2^, and stage 5 was defined as an eGFR less than 15 ml/minutes/m^2^), health insurance type, and monthly income categories using ANOVA tests or Wilcoxon test.

All tests were two-tailed, and the threshold of significance was p <0.05. The statistical analysis was performed using the packages “car”, “prettyR”, and “compare Groups”, of R software version 3.6.1 (Released 2019; R Foundation for Statistical Computing, Vienna, Austria). All necessary authorizations for this study were obtained.

Research time horizon

The analysis covered the treatment costs of patients who continued their treatment for a year at the selected healthcare facility. The time horizon adopted in the study was one year.

## Results

Population characteristics and health services utilization

Among the 88 enrolled patients, 63.6% were women and the mean age was 61.8±14.0 years. Household monthly income was less than $ 200 for more than two-thirds of participants (68.2%). Only 19.3% were affiliated with mandatory health insurance. More than half of the participants (52.3%) were in stage 4 of CKD and the CKD median duration was 47.5 (IQR: 26.2, 77.0) months. Almost two-thirds of participants (68.6%) had high blood pressure, and diabetic nephropathy was the most frequent initial nephropathy (25.0%). Tables 1-2 display the description of sociodemographic and clinical characteristics according to the CKD stage. 

**Table 1:** Sociodemographic characteristics of the study participants according to CKD stage *significant value CKD: chronic kidney disease

	Stage 3 (N=21)	Stage 4 (N=46)	stage 5 (N=21)	P-value
Age, mean±SD	60.3 ±15.2	60.7±14.1	65.8±12.1	0.325
Gender (women), n (%)	10 (47.6%)	31 (67.4%)	15(71.4%)	0.232
Marital status, n (%)				0.163
Widowed/divorced/single	3 (14.3%)	11 (24.4%)	8 (40.0%)	
Married	18 (85.7%)	34 (75.6%)	12 (60.0%)	
Number of children, median (IQR)	3.00 (2.00;6.00)	4.00 (2.00;7.00)	6.00 (4.00;9.00)	0.189
Number of dependents, median (IQR)	2.00 (0.00;4.00)	1.00 (0.00;3.00)	1.00 (0.00;2.00)	0.097
Illiterate/coranic, n (%)	14 (66.7%)	37 (82.2%)	17 (89.5%)	
Primary	5 (23.8%)	7 (15.6%)	2 (10.5%)	
Secondary/university level	2 (9.52%)	1 (2.22%)	0 (0.00%)	
Working condition, n (%)				0.284
Worker	3 (14.3%)	8 (17.8%)	2 (10.5%)	
Retired	4 (19.0%)	4 (8.89%)	0 (0.00%)	
Unemployed	14 (66.7%)	33 (73.3%)	17 (89.5%)	
Monthly income, n (%)				0.031*
< $200	18 (85.7%)	26 (57.8%)	14 (73.7%)	
> $200	3 (14.3%)	9 (20.0%)	0 (0.00%)	
Unknown	0(0.00%)	10 (22.2%)	5 (26.3%)	
Health insurance, n (%)				0.025*
Medical plan for the economically disadvantaged	14 (70.0%)	33 (75.0%)	21 (100%)	
Mandatory health insurance	6 (30.0%)	11 (25.0%)	0 (0.00%)	

**Table 2:** Clinical characteristics of the study participants according to CKD stage *significant value CKD: chronic kidney disease

	Stage 3 (N=21)	Stage 4 (N=46)	stage 5 (N=21)	P-value
Comorbidities, n (%)				
Diabetes	10 (47.6%)	15 (33.3%)	3 (15.0%)	0.077
Cardiovascular disease	8 (38.1%)	10 (22.7%)	4 (21.1%)	0.361
High blood pressure	14 (66.7%)	31 (68.9%)	14 (70.0%)	0.972
Rheumatological disease	5 (23.8%)	9 (20.0%)	4 (20.0%)	0.947
History of Surgery	8 (38.1%)	19 (42.2%)	7 (35.0%)	0.877
Tobacco (non-smoker), n (%)	15 (71.4%)	38 (84.4%)	16 (84.2%)	0.809
Initial nephropathy, n (%)				0.248
Diabetic nephropathy	1 (4.76%)	13 (28.3%)	8 (38.1%)	
Glomerulonephritis	7 (33.3%)	8 (17.4%)	5 (23.8%)	
Hypertensive nephropathy/vascular nephropathy	7 (33.3%)	9 (19.6%)	5 (23.8%)	
Dominant polycystic kidney disease/obstructive nephropathy/others	5 (23.8%)	13 (28.3%)	3 (14.3%)	
Renal biopsy (Yes), n (%)	6 (28.6%)	12 (26.1%)	7 (36.8%)	0.761
Disease duration, median (IQR)	48.0 (28.5;65.0)	42.5 (24.0;68.2)	52.0 (30.0;84.0)	0.681
Hemoglobin(g/dl), median (IQR)	9.85 (8.85;11.1)	11.5 (10.4;12.0)	12.4 (11.1;13.7)	<0.001*
Hospitalization duration in days, median (IQR)	2.00 (0.00;16.5)	0.00 (0.00;17.5)	3.00 (0.00;10.0)	0.589
Number of visits at the university hospital, median (IQR)	9.00 (4.75;12.8)	7.00 (4.00;16.5)	4.00 (3.00;5.00)	0.201
Medication, n (%)				
Angiotensin II receptors blockers	6 (28.6%)	16 (34.8%)	6 (28.6%)	0.860
Angiotensin-converting enzyme inhibitor	7 (33.3%)	10 (21.7%)	5 (23.8%)	0.590
Calcic inhibitors	3 (14.3%)	16 (34.8%)	15 (71.4%)	0.001*
Diuretics	7 (33.3%)	20 (43.5%)	7 (33.3%)	0.708
Calcium	1 (4.76%)	10 (21.7%)	4 (19.0%)	0.264
Oral Iron	2 (9.52%)	10 (21.7%)	11 (52.4%)	0.004*

Direct medical cost of CKD

Specialized Visits and Hospitalization

The mean number of hospitalizations during the study time horizon was 1.04 (95%CI: 0.71 -1.36), with a mean duration of hospitalizations was 11.3 days (95%CI: 5.27-17.3). Its related cost was estimated at $ 648.51 (95%CI: $288.67-1008.35) which counted for 32.2% of the direct medical cost. The mean number of daily care days in the hospital was 1.56 days (95%CI: 0.80-2.27), and the mean cost was estimated at $ 18.26 (95%CI: $ 9.73-26.79), which counted for 0.09% of the direct medical cost.

The majority of our patients were followed in the university hospital outpatient department. The median number of specialist visits in the university hospital was 6.00 (IQR: 4.00, 11.8) and only 10% of them had recourse to private services during the study time horizon. The mean cost of outpatient visits in the university hospital was $ 90.72 (95%CI: $ 66.10-115.34), which represents 4.4% of the direct medical cost.

Diagnosis Costs

The mean diagnosis cost of CKD including all the exploratory exams, laboratory tests, and specialized acts like biopsies was estimated at $ 605.80 (95%CI: $ 517.73-693.87), which represents 29.7% of the direct medical cost. The main component of the diagnostic cost were laboratory tests and radiology. The mean cost of laboratory tests was $ 519.05 (95%CI: $450.15-587.96) (cost in hospital was $ 325,47 (95%CI: $260.71-390.23) and in private practice was $ 190.06 (95%CI: 136,00-244,14 US$)), the mean cost of radiology exams was $ 75.86 (95%CI: $ 43.05-108.68).

Treatment Costs

More than 99% of patients were under antihypertensive treatment; the most frequent were angiotensin II receptors blockers (31.8%), followed by calcic inhibitors (38.6%), then diuretics (38.6%). Their mean costs were $ 207.64 (95%CI: $165.99-249.30). With regard to supplementation drugs, 16.6% were taking calcium, 16.6% were taking vitamin D, 26.4% were taking oral iron, 4.16% were taking intravenous iron, and 6.94% were taking erythropoietin. The mean cost of this category of drugs was $ 112.61 (95%CI: $45,29-179,93). The mean cost of chemotherapy was $ 213,65 (95%CI: $ -2.72-430.03).

The mean cost of treatment including all the drugs as well as treating acts like acute dialysis was estimated at $ 656.04 (95%CI: 398.03-914.05), which represents 32.2% of the direct medical cost. The mean cost of drugs bought by the patients was $ 425.04 (95%CI: 337.35-512.73), which represents 64.7% of the treatment cost, and the mean cost of drugs provided by the hospital was $ 231 (95%CI: $10.88-451.13), which represent 35.3% of the treatment cost.

Direct Medical Cost

Overall, the mean direct medical cost was estimated at $ 2008.80 (95%CI: $ 1528.28-2489.31x). Two-thirds of the costs were derived from the use of public resources, and the other third of the costs ($ 618.58) corresponded to out-of-pocket expenditures initially paid by the patients. The comparison of the costs between CKD stages and the health insurance category did not show any significant difference in terms of direct medical cost (Tables 3-4).

**Table 3:** Unadjusted mean annual direct medical cost per patient by cost component and by CKD stage (in $). $: United States Dollar

	Stage 3	Stage 4	Stage 5	P-value
	Mean (95%CI)	Mean (95%CI)	Mean (95%CI)	
Hospitalization	1019.81 (365.46-1674.16)	552.42 (357.43-747.40)	437.91 (296.74-579.07)	0.482
Daily hospital care	14.19 (8.05-20.33)	14.41 (6.83-21.99)	30.91(19.10-42.72)	0.270
Specialist visits at the university hospital	58.52 (37.08-79.96)	106.48 (80.28-132.68)	77.0 (57.05-96.95)	0.201
Diagnostic exams	477.62 (409.11-546.13)	656.81 (566.39-747.23)	546.0 (459.23-632.85)	0.249
Laboratory tests	399.96 (349.24-450.68)	573.10 ( 499.37-646.83)	454.63 (395.20 -514.06)	0.087
Treatment	417.56 (362.12 -473.00)	845.35 (507.87 -1182.83 )	397.32 (334.62-460.02)	0.232
Total	1993.97 (1321.14- 2666.80)	2182.18 (1725.12 -2639.24)	1490.28 (1316.37-1664.19)	0.513

**Table 4:** Unadjusted mean annual direct medical cost per patient by cost component and by health insurance type (in $). The third category of health insurance was discarded because of the small number of patients $: United States Dollar

	Medical plan for the economically disadvantaged (N=68)	Mandatory health insurance (N=17)	P
	Mean (95%CI)	Mean (95%CI)	
Hospitalization	626.34 (241.97- 1010.71)	701.14 (461.47-940.81)	0.835
Daily hospital care	20.68 (11.35-30.01)	11.77 (6.85-16.69)	0.262
Specialist visits	84.04 (62.90-105.18)	93.17 (62.97-123.37)	0.809
Diagnostic exams	608.19 (516.72-699.66)	532.51(470.39-594.63)	0.404
Laboratory tests	508.31(438.37-578.25)	498.52 (440.56-556.48)	0.902
Treatment	563.42 (356.50- 770.34)	991.65 (600.02-1383.28)	0.374
Total	1906.3 (1431.42-2381.18)	2343.55 (1844.70-2842.40)	0.502

A comparison between the costs and the monthly income categories showed a significant difference in hospitalization cost (p=0.01), and in direct medical cost (p=0.02) as shown in Table 5.

**Table 5:** Unadjusted mean annual direct medical cost per patient by cost component and by monthly income (in $). *significant value $: United States Dollar

	< $200 (N=58)	> $200 (N=12)	Unknown (N=15)	P
	Mean (95%CI)	Mean (95%CI)	Mean (95%CI)	
Hospitalization	473.55 (319.72-627.38)	2112.44 (1240.01- 2984.87)	197.67 (115.54- 279.80)	0.01*
Daily hospital care	20.46 (10.87- 30.05)	22.22 (14.25-30.19)	10.26 (4.23- 16.30)	0.67
Specialist visits	79.64 (55.72- 103.56)	72.16 (55.95-88.37)	145.75 (115.72-175.78)	0.12
Diagnostic exams	559.13 (479.06-639.20)	708.40 (616.33- 800.47)	600.71 (484.98-716.44)	0.53
Laboratory tests	482.90 (419.34-546.46)	615.56 (545.43- 685.69)	495.22 (406.99-583.45)	0.44
Treatment	714.23 (409.69-1018.77)	708.18 (569.72- 846.64)	337.48 (276.89-398.07)	0.56
Total	1852.95 (1451.52-2254.38)	3623.29 (2742.82-4503.76)	1299.98 (1135.72-1464.24)	0.02*

## Discussion

The present cost-of-illness study is the first one to assess the direct medical cost of CKD before RRT in the kingdom of Morocco. Its main findings showed that the direct medical cost of this disease in the Moroccan context is $ 2008.80 allocated mostly to hospitalization, diagnosis, and treatment. The absence of significant difference between stages is probably due to the small number of patients in stages 3 and 5 compared to stage 4. The significant differences found between costs according to monthly income for direct medical costs could be explained by the fact that private services if used for some benefits like medication and laboratory tests, are out-of-pocket expenses and are certainly more consequent for people with low monthly incomes.

This study can be compared with a Lebanese one [19], since this country is also classified as a low-middle-income country. They found that principal expenses in 102 non-dialyzed CKD patients were medication and blood tests. The median medical cost per year of non-dialysis CKD patients in Lebanon was $ 2988 (95%CI: $ 1884.15-4188.28), which is close to our findings.

The Lebanese report revealed a great percentage of non-dialyzed CKD patients paying out-of-pocket for their disease treatment and follow-up, while in our study two-thirds of expenses are paid by public services. Unlike our study, the Lebanese study found a significant difference in costs between CKD stages probably because they placed patients in stages 4 and 5 in the same group.

The economic situation of CKD in Morocco

The increase in the prevalence of CKD with its risk factors and comorbidities such as diabetes and cardiovascular diseases as well as the aging of population makes CKD a major issue for healthcare systems [20]. In Morocco, CKD and especially ESRD were already considered major health problems in 2004. It has been the subject of a political commitment and mobilization of health authorities, the civil community, and the private sector [21]. Thereby, according to its annual report on mandatory health insurance published in 2018, the Moroccan National Agency of Health Insurance, spend a third of its expenses (31.5%) to support drug purchase, and more than half of it (51.5%) is carried out by persons with long-duration afflictions, mainly ESRD (27.4%). Indeed with a prevalence of 2.9% [14], the total number of patients is around 1,075,000, while the latest statistics of the Moroccan Society of Nephrology showed that the number of dialyzed patients was 32,250 in 2020.

In Morocco, a reform process to establish universal health coverage was launched in 2002. Law on basic medical coverage [22] launched a series of health financing reforms to establish universal health coverage through a subsidized SHI scheme (medical plan for the economically disadvantaged), under which poor people make no contributions, the vulnerable make small contributions, and all others would be covered by mandatory health insurance schemes.

In 2012, the Moroccan Ministry of Health and the French Agency of Biomedicine conducted an analysis of the cost of ESRD. Their final report shows that the hemodialysis costs were $ 27,515.89 per year per patient, and the transplantation costs were $ 38,258.79 the first year and then $ 9416 per year per patient. Also, the out-of-pocket expense for patients was estimated at $ 792.87 per year for transplantation and $ 897.27 per year for dialysis. Finally, this analysis estimated that the benefit to society after 15 years was $ 181,611.80 for people who underwent transplantation rather than hemodialysis.

The cost of CKD in the world

Several studies around the world estimated the cost of CKD before the start of RRT. An Italian cross-sectional cost-of-illness study by Turchetti et al. included all adult outpatients in the 14 main Nephrology Centers of the Tuscany Region for seven weeks in 2012-2013 [10]. Using an approach close to ours, the direct medical costs were estimated using tariffs for laboratory tests, diagnostic exams, visits, hospitalization, and the price of drugs. A total of 279 patients in stage 4 and 205 patients in stage 5 were enrolled. The estimated mean annual social cost of a patient with CKD was € 7422 (± €6255) for stage 4 and € 8971 (± €6503) for stage 5 (p <0.05). Direct medical costs were higher in stage 5 as compared to stage 4. Overall, the mean cost of CKD before RRT in Italy was € 8077.80; the drugs represented 28.6% and the indirect costs represented 37.3% of total costs [10].

A German study by Gandjour et al. took the perspective of the German Statutory Health Insurance (SHI) system and analyzed claims data on 3,687,015 insured patients from the year 2014 [9]. The average cost per person per year in an age- and a gender-matched control group of the normal population were € 2,876 (95%CI: € 2,798-2,955) and ≥ 2.8-fold higher in CKD patients (€ 8,030; 95%CI, €7,848-8,212) at CKD stage 3, € 9,760 (95%CI: € 9,266-10,255) at CKD stage 4, and € 44,374 (95%CI: €43,608-45,139) on dialysis. At stages 3 and 4 of CKD, the major cost driver was hospitalizations, contributing to more than 50% of total expenditures. In our study, the percentage of hospitalization contribution was estimated at 32.2% of the direct medical cost.

In Canada, the population-based cohort conducted by Manns et al. in 2014, included 219,641 adults with CKD not dialyzed nor transplanted, using administrative health data [23]. The primary outcome was one-year cumulative unadjusted healthcare costs, including the cost of drugs, physician visits, emergency department visits, outpatient procedures, and hospitalizations for the year following each patient’s index date. The mean unadjusted cumulative one-year cost of care was Canadian dollar (Can$) 14,634 per patient (Can$ 1496, Q3 = Can$ 10,221). Costs were higher for patients with more comorbidities, lower eGFR, and more severe albuminuria.

The American study by Smith et al. conducted on 13,796 persons with CKD and their age- and gender-matched controls showed that the total per-patient follow-up costs were $ 38,764 for cases (95%CI: $37,033-40,496) and $ 16,212 for controls (95%CI: $15,644-16,780) in stage 2, $ 33,144 for cases (95%CI: $32,578-33,709) and $ 18,964 for controls (95%CI: $18,730-19,197) in stage 3, and $41,928 for cases (95%CI: $39,354-44,501) and $19,106 for controls (95%CI: $18,212-20,000) in stage 4 [11].

The comparison between those studies and the current one is not easy since they all concerned high-income countries and their methodology are different from ours. Thus, except for the Italian cost-of-illness study, the others used an incidence approach and a cohort design, and two compared the cost between patients with and without CKD [11,24].

Strengths and limitation 

This study is a cost-of-illness study on CKD before RRT, conducted in a middle-income country while, to our knowledge, most of the studies conducted in countries like ours were exclusively on dialysis cost [25,26]. We tried to be as transparent as possible in setting out data sources and key assumptions since we are perfectly aware that the accuracy of cost estimates is inevitably dependent on the quality of underlying data sources and the appropriateness of the assumption made in economic modeling [5]. The third strength of this study is that we did not use any restrictions regarding comorbidities [8].

The first limitation of this study is that it reports the data from only one of the 12 regions of Moroccan territory. Even if the enrolment took place in a university hospital that receives patients from all around the studied region, the representability of the sample is still uncertain due to the heterogeneity of Moroccan regions. The second limitation was that the costs for people without CKD were not included in this study, which made the assessment of the incremental costs associated with CKD impossible [22]. Finally, only the number of nephrology visits was surveyed because it’s hard to attribute the elevation of the number of other visits like endocrinology and cardiology to CKD.

## Conclusions

The cost of CKD in its early stages is still lower than the cost of dialysis or transplantation in Morocco. This cost is mostly divided between hospitalization, diagnosis, and treatment. This study's findings bring to light the necessity of primary prevention in patients with high blood pressure and diabetes to avoid the onset of CKD and the secondary prevention of patients in stages 4 and 5 of CKD to postpone or even prevent the progression to ESRD. Also, screening campaigns could be set for an early diagnosis of patients suffering from CKD. 
